# Recent advancements in diffusion MRI for investigating cortical development after preterm birth—potential and pitfalls

**DOI:** 10.3389/fnhum.2014.01066

**Published:** 2015-01-21

**Authors:** J. Dudink, K. Pieterman, A. Leemans, M. Kleinnijenhuis, A. M. van Cappellen van Walsum, F. E. Hoebeek

**Affiliations:** ^1^Department of Neonatology, Pediatric Intensive Care and Pediatric Radiology, Erasmus Medical Center - Sophia Children’s HospitalRotterdam, Netherlands; ^2^Image Sciences Institute, University Medical Center UtrechtUtrecht, Netherlands; ^3^Oxford Centre for Functional Magnetic Resonance Imaging of the Brain, University of OxfordOxford, UK; ^4^Department of Anatomy, Donders Institute for Brain, Cognition, and Behaviour, Radboud University Medical CenterNijmegen, Netherlands; ^5^Department of Neuroscience, Erasmus Medical Center RotterdamRotterdam, Netherlands

**Keywords:** diffusion magnetic resonance imaging, DTI, cortical imaging technique, prematurity, cortical development, cortical development and plasticity, diffusion MRI

## Abstract

Preterm infants are born during a critical period of brain maturation, in which even subtle events can result in substantial behavioral, motor and cognitive deficits, as well as psychiatric diseases. Recent evidence shows that the main source for these devastating disabilities is not necessarily white matter (WM) damage but could also be disruptions of cortical microstructure. Animal studies showed how moderate hypoxic-ischemic conditions did not result in significant neuronal loss in the developing brain, but did cause significantly impaired dendritic growth and synapse formation alongside a disturbed development of neuronal connectivity as measured using diffusion magnetic resonance imaging (dMRI). When using more advanced acquisition settings such as high-angular resolution diffusion imaging (HARDI), more advanced reconstruction methods can be applied to investigate the cortical microstructure with higher levels of detail. Recent advances in dMRI acquisition and analysis have great potential to contribute to a better understanding of neuronal connectivity impairment in preterm birth. We will review the current understanding of abnormal preterm cortical development, novel approaches in dMRI, and the pitfalls in scanning vulnerable preterm infants.

## Introduction

During the final trimester of pregnancy, the human brain rapidly develops by a complex interplay of genetic, epigenetic and environmental factors. Preterm infants are born during this period that is critical for neuronal connectivity, brain growth and cortical maturation, which together puts them at risk for functional impairments that are likely to persist into later life (Saigal and Doyle, 2008; Volpe, 2009). In particular the development of the cerebral cortex is imperative for the social and emotional well-being (e.g., Salmaso et al., 2014). Histological studies in both animals and humans have demonstrated that white and gray matter develop in close relation, and that isolated white matter (WM) injury sparing the cerebral cortex is uncommon (Govaert et al., 2006; Pierson et al., 2007; Sizonenko et al., 2007; Volpe, 2009; Okabayashi et al., 2011). Diffusion-MRI (dMRI) is a valuable tool to study brain development *in vivo*, and is based on its ability to characterize diffusion patterns of water molecules within the brain (Basser et al., 1994). As these patterns are directly related to brain microstructure, different models have been developed to characterize the diffusion signal in detail. The diffusion tensor (DT) model has been commonly used for this purpose, and represents the diffusion characteristics within each voxel as an ellipsoid (Basser et al., 1994; Jones and Leemans, 2011). Using only six parameters, this model is able to provide a quantitative description of the diffusion characteristics representing neuroanatomy. The ellipsoidal shape is more elongated (cigar-shaped) in regions with highly aligned and densely packed tissue components, such as the corpus callosum, representing *anisotropic* diffusion. In contrast, the DT shape resembles a sphere in brain regions with less aligned fiber tissue, wherein the diffusion pattern is called *isotropic*. Several studies have pointed out that the DT model is less adequate in representing complex fiber orientations within a single voxel (Tournier et al., 2004, 2011; Jeurissen et al., 2013), which is furthermore reflected by the fact that diffusion tensor imaging (DTI) has been predominantly used to characterize large WM bundles such as the corpus callosum and pyramidal tract but hardly to study “complex” brain regions containing crossing fibers and more dispersedly organized microstructure such as gray matter (Martinussen et al., 2005; Pannek et al., 2014).

Present-day, neuroimaging studies resulted in the development of acquisition and processing pipelines designed to derive more detailed information about the precise diffusion characteristics within each voxel. These advances increase the applicability of dMRI to regions of more complex microstructure such as the cerebral cortex. Several higher-order models to fit the diffusion data have been developed recently, including models designed to study cortical microstructure, such as *neurite orientation dispersion and density imaging* (NODDI; Zhang et al., 2012). This mini-review will briefly outline these and other recent developments regarding cortical imaging and discuss related challenges and pitfalls.

## Insights in impaired cortical development from human and animal specimen studies

For long, histological substrates of brain damage following preterm birth were most prominently characterized by large, focal WM lesions, adjacent to the ventricles, known as periventricular leukomalacia (PVL). Although cystic changes of WM are most prominent in PVL, several studies have outlined that relative sparing of the cerebral cortex is implausible in this type of WM injury. Andiman et al. (2010) histologically assessed the cortical microstructure in specimens of human tissue collected following focal periventricular WM injury possibly induced by preterm birth, and observed a significant reduction in pyramidal neuron density in layer V of the overlying cerebral cortex, indicating cell death of cortical neurons in focal WM injury (Andiman et al., 2010). A commonly used middle cerebral artery stroke model in mice showed comparable histological features, such as the histological abnormalities in layer V of the cortex. This included both morphological alterations of cell components and signs of neuronal necrosis (de Oliveira et al., 2014). In addition, subcortical nuclei were also affected in PVL cases, showing significant neuronal loss in thalamus, globus pallidus and the cerebellar dentate nucleus (Pierson et al., 2007). These findings exemplify that opposed to isolated WM injury, impaired neurodevelopment in preterm infants represents a complex interplay between gray and WM damage, leading to structural changes throughout the entire brain (Tymofiyeva et al., 2012; Ball et al., 2013a).

With the improved spatial resolution of state-of-the-art radiological techniques (e.g., ultra-high field MRI) the neonatal care specialists also encounter diffuse anatomical anomalies that affect both cortical and subcortical structures. Often these subtle aberrations are found in preterm children that suffered from hypoxia, ischemia or inflammation. Although the extent of such anatomical aberrations seems limited when visualized using conventional imaging, both human imaging and histology studies have demonstrated that these changes in the preterm brain can nevertheless significantly impact on long-term functional outcome (Ment et al., 2009). Moreover, using an experimental sheep model it was recently shown that the cortical development was severely affected following mild levels of ischemia, which evoked non-cystic and thus diffuse WM alteration (Dean et al., 2013). Using *ex vivo* high-field diffusion MRI it was also shown that normal cortical development was impeded, i.e., the physiological decrease in cortical anisotropy resulting from emerging cellular complexity during development was less pronounced in sheep exposed to moderate ischemia.

In contrast to focal WM changes such as PVL, the number of cortical neurons was not reduced in this type of WM damage, but Golgi-staining showed that ischemia induced a significant reduction of dendritic branching in the cerebral cortex, which could well explain the observed differences in anisotropy compared to age-matched controls. These results indicate that disrupted cortical development in moderate ischemia does not have to be linked to drastic effects like neuronal loss, but can also evoke “milder” effects like morphological aberrations (Dean et al., 2013).

Results of such translational studies of impaired cortical development are very useful, as they contribute to ensuring correct interpretation of *in vivo* human imaging results. By combining histology with MR-imaging in animal models and specimen studies, histological features of disrupted cortical development can be correlated to MR abnormalities seen in preterm born infants. Histology of the human cortex has been used as validation of normal brain cortical architecture (Kleinnijenhuis et al., 2013b; White et al., 2013), as well as to assess how histopathological findings correlate to cortical dMRI (Hulst and Geurts, 2011; Kolasinski et al., 2012; Gao et al., 2013; Leigland et al., 2013). These insights can be expected to help develop reliable, non-invasive, *in vivo* neuroimaging biomarkers for early prediction of impaired neurodevelopment.

## MRI studies of impaired cortical development in humans

The shift to more subtle radiologic abnormalities in both cortex and WM demands new ways to assess structure and development of the preterm brain. dMRI can provide additional insights in the characteristics of the preterm brain by mapping the diffusion pattern of water molecules. Because tissue components hinder the random motion of water molecules, dMRI measurements are directly related to underlying tissue microstructure (Basser and Jones, 2002; Mori and Van Zijl, 2002). Analysis of preterm brain damage using dMRI can thereby contribute to a better understanding of injury-mechanisms underlying impaired neurodevelopment, by revealing alterations in neuronal organization and extracellular matrix composition (Basser and Jones, 2002; Mori and Van Zijl, 2002).

Conventional T1 imaging has been used to assess the impact of preterm birth on cortical development. This sequence is particularly suitable for assessment of cortical volume and surface area, hence it provides good contrast between white and gray matter in combination with high spatial resolution. Studies making use of this have shown that cortical volume and surface area are substantially affected by preterm birth. Phillips et al. (2011) showed that in early childhood, cortical thickness was significantly higher and surface area significantly lower among preterm born infants than in term born controls (Phillips et al., 2011). This indicates that the normal pattern of cortical maturation—a combination of cortical thinning and surface area expansion—is substantially delayed or disrupted by preterm birth. The importance of these findings is highlighted by the fact that impaired cortical growth between 24 weeks and term-equivalent age is directly related to neurocognitive abilities in later life (Rathbone et al., 2011). The degree of cortical folding seems directly related to the extent of WM connectivity (Melbourne et al., 2014), which is particularly relevant because studies in ex-preterm adolescents and adults show that characteristic changes in cortical folding, thickness and volume persist in later life (Martinussen et al., 2005; Nagy et al., 2011; Skranes et al., 2013).

## Advanced diffusion weighted imaging techniques

Cortical diffusion MRI can help to determine *in vivo* how changes in cortical folding and thickness are related to cortical changes at a cellular level. Advances in dMRI, such as more widespread application of high-angular resolution diffusion imaging (HARDI), enable a more reliable assessment of cortical microstructure (Tuch et al., 2002). Benefits of HARDI acquisition arise from a substantial increase in number of diffusion-encoding directions, providing a more reliable and extensive characterization of the 3d diffusion profile. Next to an increase in angular resolution, increased spatial resolution is desirable, because it facilitates more reliable differentiation among cortical regions, underlying WM and surrounding cerebrospinal fluid. Higher-order processing algorithms of HARDI-data provide more detailed information than the tradition DT model, as these models aim at extracting the diffusion characteristics from each voxel more extensively.

**Figure 1:**
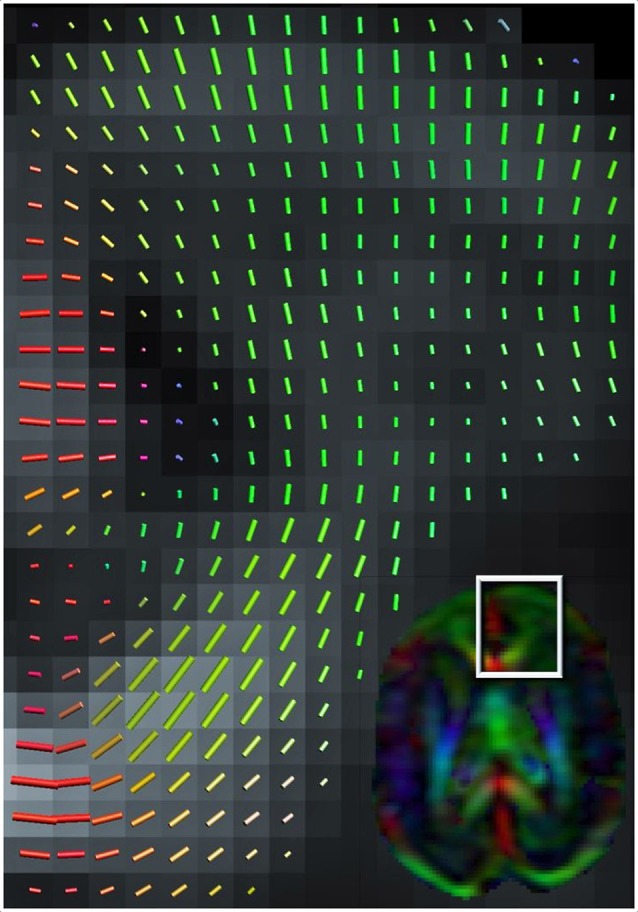
**Color-coded orientation map of a preterm infant’s brain (born at 26 weeks of gestation and scanned at 30 weeks gestation at 1.5 Tesla MRI scanner) calculated from diffusion tensor imaging**. Each pixel contains information about the eigenvector (Green color signifies preferential diffusion in the antero-posterior direction; Red in the latero-lateral direction; Blue in the superior-inferior direction) and extent of diffusion anisotropy (intensity of color) which summarizes the information obtained from each 3 × 3-matrix tensor. The cortex at 30 weeks gestation still has a clear radial organization (shown in close-up).

The structure of the neocortex dramatically increases in complexity during development (McKinstry et al., 2002; Ball et al., 2013b). During developmental stages characterized by high densities of radial glial fibers, the DT model might be an adequate model for describing diffusion in the cortex (Figure 1). Furthermore, structural development of the cortex can be detected with the DT model (Neil et al., 1998; McKinstry et al., 2002), but the model’s specificity is limited, because multiple features of the microstructure have similar effects on tensor characteristics (Vos et al., 2011, 2012). For example, the decrease in anisotropy observed over cortical development is thought to results from emerging dendritic arborization (Dean et al., 2013), but the same anisotropy decrease could also result from, for instance a reduction in radial glial fibers (Sizonenko et al., 2007). Most likely, these different changes happen concurrently and therefore it is essential to distinguish these contributions and other microstructural features, not only with postmortem techniques, but also with *in vivo* methods such as diffusion MRI.

**Figure 2:**
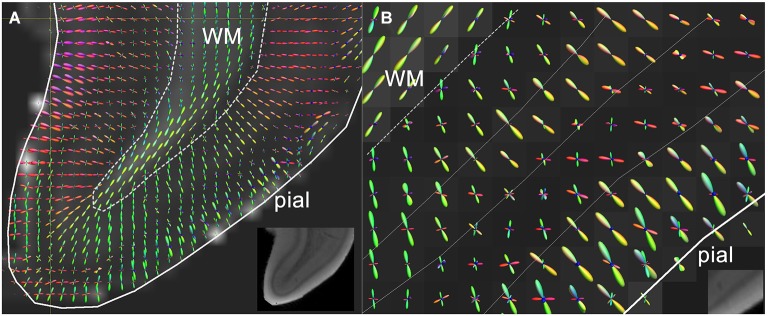
**(A)** High resolution diffusion-MRI of sub-cortical white matter and cortical gray matter of the adult human primary visual cortex. High angular and spatial resolution data (768 directions at 300 micrometers isotropic resolution) was used to generate orientation distribution functions (ODF’s) for each voxel. The spikes of the ODF are color-coded and represent the different directions of orientation within a single voxel. The ODF’s in the cortex are mainly orientated perpendicular to the cortical surface, and represent its radial organization, whereas underlying white matter is arranged corticofugally. **(B)** Close-up of the cortex in a neighboring slice, showing that the ODF’s are able to discern different layers of the cortex based on differences in microstructural characteristics between different laminae. The fractional anisotropy image is used as a backdrop image. Greyscale insets represent the anatomical GRE reference images showing cortical layers including the stria of Gennari. Adapted from Kleinnijenhuis et al. (2011).

As mentioned above, appealing alternatives to the DT model are available to capture more of the cortical complexity. The limitation of the DT model of describing diffusion behavior as an ellipsoid can be overcome by fitting models that allow more peaks in the modeled diffusion profile (Frank, 2002; Jansons and Alexander, 2003; Tuch et al., 2003; Tournier et al., 2004; Wedeen et al., 2005). By making use of high-angular resolution data, it is possible to fit more complex models which include multiple fibers. Such models have been widely adopted for resolving crossing fiber populations in WM tractography (Jeurissen et al., 2011, 2014; Pannek et al., 2014; Tax et al., 2014a). Likewise, complex cortical fiber arrangements emerging during gestation should also be reflected in the fiber orientation distributions derived from these data. Metrics specific to various cortical fiber populations might then be derived for application of this method in WM of the preterm brain (Raffelt et al., 2012; Dell’Acqua et al., 2013). Although radial and tangential components in the fiber orientation distribution have been *ex vivo* demonstrated (Figure 2) in adult human (Kleinnijenhuis et al., 2013a) and animal cortex (Dyrby et al., 2011), the performance of these models under such anisotropic conditions is uncertain (Parker et al., 2013). Furthermore, because scan time increases linearly with the number of directions, *in vivo* application of these elaborate sampling schemes in preterm newborns is particularly challenging.

An innovation that could also be valuable for investigating the cortex in preterm infants is the estimation of quantitative measures of tissue microstructure. This approach surpasses the measures obtained from the DT model, as it can directly inform on the fraction, size and shape of various tissue compartments. Whereas extracellular water is—although hindered in its path—free to diffuse throughout the extracellular space, intracellular water is restricted within the bounds of the cell. Sensitization to these different water pools is achieved by varying the b-value (or diffusion time), where higher b-values represent to some extent the intracellular signal, increasing the specificity to the tissue structures (e.g., Assaf and Basser, 2005). This simple principle has been used in several investigations of the preterm brain (Dudink et al., 2008; Ferizi et al., 2014; Pannek et al., 2014; Riffert et al., 2014). The diffusion kurtosis tensor (Jensen et al., 2005) is a somewhat more elaborate model that allows separation of the Gaussian and non-Gaussian contribution to the diffusion attenuation, assumed to represent the extracellular and intracellular spaces. A recent report demonstrates a marked decrease of radial kurtosis in the third trimester (Jeon, 2014), suggesting that it is a sensitive measure for disruption of radial glial fibers. Although the diffusion kurtosis isolates non-Gaussianity in the signal, it does not inform on the source of this non-Gaussianity. This is particularly relevant for the developing cortex, because non-Gaussianity can also result from fiber dispersion. The biophysical multi-compartment model NODDI (Zhang et al., 2012) is able to account for and inform on fiber dispersion, by explicitly including it in the signal model. The main parameters that can be estimated from the NODDI model are intracellular volume fraction and the orientation dispersion index. The first reports applying this model to the preterm brain (Eaton-Rosen, 2014; Kunz et al., 2014), suggest an increase in orientation dispersion during the third trimester, while the intracellular fiber volume fraction does not change.

## Data quality and patient safety

Although present-day and future innovations of dMRI offer new ways to increase understanding of disturbed cortical development in preterm infants, obtaining good quality images is particularly challenging in this patient group. For example, higher heart- and breathing rates in preterm infants and tendency of head movement during scanning can easily result in poor data quality, as dMRI sequences are motion-sensitive and artifact-prone. These matters are particularly relevant when studying cortical microstructure using these techniques, as certain types of distortions are more pronounced along the rim of brain tissue (Jones and Cercignani, 2010). Awareness of acquisition and processing steps determining data quality is therefore essential. To start with, fine-tuning of MR acquisition to the specific characteristic of the preterm brain and infant is desirable. Neonatal scanning hardware, such as neonatal coils, MR-compatible incubators, fixation pillows and noise-reducing earplugs have the potential to increase image quality and patient safety during scanning (Pannek et al., 2012). Furthermore, as neonatal dMRI data are highly sensitive to cardiac pulsation artifacts, the introduction of pulse triggering might benefit dMRI data quality considerably (Kozák et al., 2013). Still, extensive assessment of data quality is essential to ensure reliability of images. An approach combining visual inspection of raw diffusion data with software-based quality checks seems essential to ensure data reliability, as certain types of artifacts can hardly be seen on the diffusion data themselves (Tournier et al., 2011; Heemskerk et al., 2013). Dependent on the extent of quality loss, datasets need to be excluded from analysis or optimized using artifact and motion correction software in order to prevent inclusion of erroneous diffusion metrics in study results (Chang et al., 2005; Veraart et al., 2013; Collier et al., 2014; Plaisier et al., 2014; Tax et al., 2014b). This especially holds true for advanced neonatal diffusion MRI sequences, as complex acquisition and processing pipelines are accompanied with many pitfalls (Jones and Cercignani, 2010; Kersbergen et al., 2014).

## Conclusion

High resolution dMRI has great potential to improve understanding of cortical neuronal connectivity impairment in children born preterm. During the fetal period, axons from and to the cortical plate also start to form, and tends to align in the early phase of development. This radial structure forms the basis for the columnar organization of the fetal cortex (Sidman and Rakic, 1973; Rakic, 2002). As advances in neonatal imaging hardware and software will reduce traditional limitations of dMRI, future neonatal dMRI studies could go as far as study normal and abnormal maturation of columnar organization of our neocortex (Mountcastle, 1997). Next to that, HARDI-based imaging studies in animals and adults have shown that it is possible to discern different cortical regions based on the unique diffusion-“fingerprint” of different cortical regions. Likely, these advances will eventually result in the development of microstructure-based atlases, which can be used to study developmental patterns and eventual plasticity of individual cortical regions that are defined using microstructure (Schnell et al., 2009; Nagy et al., 2013; Wu et al., 2014; Aggarwal et al., 2015). Validation of these new techniques using histology is essential to ensure good correlation between microstructure and imaging.

## Conflict of interest statement

The authors declare that the research was conducted in the absence of any commercial or financial relationships that could be construed as a potential conflict of interest.
